# Isolation and molecular characterization of clinical and environmental dematiaceous fungi and relatives from Iran

**DOI:** 10.18502/cmm.7.3.7798

**Published:** 2021-09

**Authors:** Gholamreza Shokoohi, Hamid Badali, Bahram Ahmadi, Kazuo Satoh, Sadegh Nouripour-Sisakht, Mahnaz Nikaeen, Mohsen Gramishoar, Nilufar Jalalizand, Sahar Kianipour, Hossein Mirhendi, Koichi Makimura

**Affiliations:** 1 Department of Parasitology and Mycology, Faculty of Medicine, Jahrom University of Medical Sciences, Jahrom, Iran; 2 Zoonosis Research Center, Jahrom University of Medical Sciences, Jahrom, Iran; 3 Invasive Fungi Research Center, Faculty of Medicine, Mazandaran University of Medical Sciences, Sari, Iran; 4 Department of Medical Mycology, Faculty of Medicine, Mazandaran University of Medical Sciences, Sari, Iran; 5 Department of Medical Laboratory Sciences, Faculty of Paramedical, Bushehr University of Medical Sciences, Bushehr, Iran; 6 General Medical Education and Research Center, Teikyo University, Tokyo, Japan; 7 Cellular and Molecular Research Center, Yasuj University of Medical Sciences, Yasuj, Iran; 8 Department of Environmental Health Engineering, School of Public Health, Isfahan University of Medical Sciences, Isfahan, Iran; 9 Department of Medical Parasitology and Mycology, School of Public Health, Tehran University of Medical Sciences, Tehran, Iran; 10 0 National Institute of Health Research Isfahan Health Research Station, Tehran University of Medical Sciences, Tehran, Iran; 11 1 Department of Medical Parasitology and Mycology, School of Medicine and Reference Mycology Laboratory , Isfahan University of Medical Sciences, Isfahan, Iran; 12 2 Medical Mycology, Graduate School of Medicine, Teikyo University, Tokyo, Japan

**Keywords:** Dematiaceous fungi, Iran, ITS rDNA region, Molecular identification

## Abstract

**Background and Purpose:**

The frequency and genetic diversity of black fungi in environmental and clinical settings have not been fully studied in Iran. This study aimed to identify and evaluate intra- and inter-species DNA sequence variation and also understand the phylogenetic relationships of melanized fungi and relatives isolated from different geographical regions of Iran.

**Materials and Methods:**

In total, 111 clinical and environmental strains of dematiaceous fungi were isolated, and their internal transcribed spacer ribosomal DNA (rDNA) regions were sequenced and analyzed.

**Results:**

An inter-species nucleotide sequence diversity rate of 1 to 464 nucleotides was observed between the species. Intra-species differences were found in the strains of *Alternaria alternata, Cladosporium cladosporioides, Alternaria tenuissima, Curvularia spicifera, Aureobasidium pullulans, Curvularia hawaiiensis, Neoscytalidium dimidiatum, Alternaria terricola, Alternaria chlamydospora, Didymella glomerata,* and *Drechslera dematioidea* by 0–59, 0–22, 0–4, 0–4, 0–3, 0–2, 0–2, 0–2, 0–2, 0–1, and 0–1 nucleotide, respectively.

**Conclusion:**

The internal transcribed spacer rDNA is useful for the discrimination of several taxa of dematiaceous fungi. However, a better understanding of the taxonomy of species of *Alternaria* requires a larger rDNA region or a library of other gene sequences.

## Introduction

Dematiaceous fungi are characterized by the presence of pale brown-to-dark melanin-like pigments in the cell wall, which are linked to the pathogenicity of these fungi [ 1 , 2 ]. They comprise a large number of filamentous, yeasts, and yeast-like fungi and relatives, which are found in soil, air, wood, plant, and organic debris [ 3 ]. Numerous species in this group are known to cause cutaneous lesions and severe brain encephalitis. Besides, under suitable conditions, they may produce toxins that can pose serious health risks to humans and animals [ 1 , 4 , 5 ]. Moreover, some of these fungi are of industrial importance and may be used in the production of cellobiose dehydrogenase, citric acid, and pullulan [ 6 ]. 

Despite the increasing importance of dematiaceous fungal infections, little is known about their epidemiology, mode of transmission, or pathogenesis. Epidemiological studies of dematiaceous fungi provide awareness and accurate information on their prevalence. Furthermore, such studies help develop control strategies regarding infections caused by these fungi and improve the diagnosis and development of treatment options [ 7 ]. Correct identification to the species level is essential for epidemiological, pathological, toxicological, and industrial purposes, as well as for targeted antifungal therapy [ 7 , 8 ].

For ages, phenotypic methods, including biochemistry, morphology, and physiology have formed the backbone of the identification and taxonomy of dematiaceous fungi [ 9 , 10 ]. Due to the diversities and similarities among different species, morphological features may often be indistinct and inadequate for species identification [ 10 , 11 ]. For accurate identification of these fungi, the focus has shifted towards molecular strategies with the advantages of limited hands-on activity, less required experience, and increased reliability and reproducibility compared with conventional diagnosis [ 12 ]. 

Usage of molecular methods to provide precise and timely information for health professionals is clearly advantageous. Molecular methods, in conjunction with conventional methods or alone, have great potential to develop the analysis of dematiaceous fungi [ 13 ]. Nevertheless, various factors, such as nonspecific genetic amplification from other sources (the environment or the host gene), samples containing a mixed infection, an incomplete database particularly related to GenBank, are the limitations to well describe the epidemiology by molecular methods [ 14 ]. 

Different DNA-based techniques have been used for the identification of black fungi, including polymerase chain reaction (PCR)-restriction fragments length polymorphism, amplified fragment length polymorphism, real-time PCR, arbitrarily primed PCR, rolling circle amplification, and sequence analysis of different regions of the DNA [ 15 - 20 ]. Partial small subunit (SSU), D1/D2 domain of the large subunit, and internal transcribed spacer (ITS) of the ribosomal DNA, chitin synthase (CHS) gene, and mitochondrial DNA (mtDNA) are examples of target DNAs for sequence-based identification [ 8 , 21 - 23 ]. 

The DI/D2 domain is not a useful marker for the identification of some species that have identical sequences or an intra-species variation of less than 0.5% [ 8 ]. Likewise, sequence data of mtDNA and CHS are not available for all species in GenBank, and partial SSU sequences with little nucleotide variation make the SSU rRNA gene a relatively poor target for discrimination of these fungi [ 22 ]. In contrast, phylogenetic analysis and identification of black fungi and relatives based on sequencing of the ITS1 and ITS2 regions has shown to be useful and remains the gold standard target [ 22 ]. 

No study has been performed in Iran about the occurrence and distribution of black fungi in clinical and environmental settings. Therefore, the present research project aimed to identify and evaluate inter- and intra-species variation within, and also understand phylogenetic relationships of dematiaceous fungi isolated from different geographical parts of Iran. The preliminary data provided in this study could also be useful for improving the differentiation and diagnostic detection of black fungi in the epidemiological, clinical, environmental, and industrial settings.

## Materials and Methods

### Samples and fungi isolation

In total, 350 samples, including soil, plant, wood, organic debris, and air were randomly collected from different parts of the center, south, and southwest of Iran, i.e., Shiraz, Bushehr, Isfahan, Ahwaz, and Yasuj cities. This study was approved by the Ethics Committee of Tehran University of Medical Sciences, Tehran, Iran. 

Approximately 20 g of each sample (except for air samples) was suspended in 100 mL sterile saline containing 200 U penicillin, 200 μg/ml streptomycin, and 200 μg/ml chloramphenicol. After initial incubation at room temperature for 30 min, 20 mL of sterile mineral oil was added to the solution, followed by vigorous shaking for 5 min. The samples were left for 20 min to let the debris settle down, and the oil-water interphase was carefully collected, inoculated onto the Sabouraud dextrose agar supplemented with 50 mg/L of chloramphenicol (Merck, Germany), Mycobiotic agar (Merck, Germany), home-made potato dextrose agar, and malt extract agar (MEA; Merck, Germany). Afterward, it was incubated for up to four weeks at 28 °C in darkness. The colonies of dematiaceous fungi were then isolated and stored on MEA prior to use [ 24 ]. 

In addition, samples were obtained from bathrooms and washing machines by using sterile cotton swabs moistened with physiological saline which were transported in tubes and inoculated onto MEA agar [ 25 ]. Air sampling was performed by the settled plate method using Sabouraud dextrose agar containing chloramphenicol (100 mg/L), gentamicin (40 mg/L), homemade potato dextrose agar, and MEA [ 26 ]. Plates were located for 30 min at different heights on the ground. All plates were incubated at 28 °C for at least 4 weeks until the appearance of slow-growing dark colonies [ 27 ]. 

A variety of clinical specimens, including nail, mouth, and sinus samples were collected from patients suspected of fungal infections and submitted to four medical mycology laboratories in Tehran, Isfahan, and Ahwaz, Iran. The fungi were grown on MEA at 28 ºC followed by at least a five-day slide culture and preparation of mount in lactophenol aniline blue. The colonies were studied by observation of the macroscopic morphological features (i.e., growth rate, color, shape, size, and topography) and microscopic examination of the characteristics of the hyphae, conidiophores, conidia, and other conidiation properties [ 9 ].

### Molecular characterization

Genomic DNA was extracted from the isolated colonies using the glass-bead phenol-chloroform method as previously described [ 3 ]. The ITS rDNA regions were amplified using 0.25 μM of the fungal universal primers V9G and LS266, 12.5 μL of 2× premix (Ampliqon, Denmark), 1 μL of DNA template, and enough water to produce a final volume of a 25 μL reaction mixture. The PCR cycles consisted of preheating at 94 °C for 5 min, 30 cycles of 30 s at 94 °C, 45 s at 60 °C, and 45 s at 72 °C followed by a final extension step of 7 min at 72 °C. The PCR products were subjected to 1.5% agarose gel electrophoresis and photographed under UV irradiation [ 3 , 28 ]. 

### >Sequencing and phylogenetic analysis

The PCR products were sequenced in one direction by the primer V9G using the ABI PRISM Big Dye Terminator Cycle Sequencing Ready Reaction Kit (Applied Biosystems, Foster City, CA, USA) on an automated DNA sequencer (ABI PrismTM3730 Genetic Analyzer, Applied Biosystems) according to the instructions of the manufacturer. The obtained sequence data were imported into MEGA software (version 6), ambiguous regions were edited manually to improve alignment accuracy, and final identification of isolates was performed by comparing the obtained sequences with the reference sequences of the National Center for Biotechnology Information database. 

Sequences were subjected to BioEdit software (version 7.0.5) for pairwise comparisons and multiple alignments to determine intra- and inter-species similarities and differences in nucleotides. The Maximum Likelihood method was applied to the phylogenetic analysis using unambiguously aligned sequences with the Tamura-Nei parameter with substitution model as implemented in the MEGA software (version 7) [ 29 ]. Bootstrap values equal to or greater than 70% were considered significant. 

## Results

Clinical and environmental strains were collected during a 2-year period. In total, 111 strains of potential melanized fungi were isolated out of 350 samples collected from soil, air, and other different environmental sources. Colony characteristics of each colony were studied and subjected to species identification based on ITS-rDNA sequencing. 

The PCR yielded a single band of approximately 950-1000 base pair (bp) on gel electrophoresis. Based on DNA sequencing, the clinical strains (n=9) comprised *Alternaria alternata* (n=1), *Alternaria malorum* (n=2), *Neoscytalidium dimidiatum* (n=2), *Neoscytalidium novaehollandiae* (n=1), *Aureobasidium pullulans* (n=1), *Curvularia hawaiiensis* (n=1), and *Cladosporium sphaerospermum* (n=1) (Table 1). 

**Table 1:** Summary of characterization, source, and identification of dematiaceous fungi isolated from clinical specimens.

No.	Age/gender/year	Source	City	Direct examination	Isolated on culture	Accession nr.
1	36/F/2016	Sinus discharge	Tehran	Mycelium elements	*Alternaria alternata*	KY788023
2	53/M/2016	Sinus discharge	Isfahan	Mycelium elements	*Alternaria malorum*	KY788040
3	27/M/2013	Skin lesion	Isfahan	Mycelium elements	*Alternaria malorum*	JQ219160
4	57/F/2015	Sinus discharge	Tehran	Mycelium elements	*Neoscytalidium dimidiatum*	KY788092
5	49/F/2015	Nail	Tehran	Mycelium elements	*Neoscytalidium dimidiatum*	KY788091
6	52/F/2016	Nail	Tehran	Mycelium elements	*Neoscytalidium novaehollandiae*	KY788097
7	55/M/2014	Nail	Ahwaz	Mycelium elements	*Aureobasidium pullulans*	KY788108
8	65/M/2015	Mouth lesion	Tehran	Mycelium elements	*Curvularia hawaiiensis*	KY788102
9	65/F/2014	Nail	Tehran	Mycelium elements	*Cladosporium sphaeroespermum*	KY788060

The mean age and age range of patients were 51 and 27-65 years, respectively. Most patients were female (56%) and in the majority of cases, the infection had been present for a long time. In mycological tests, the characteristic mycelium was seen in direct examination of all nine specimens (Table 1). The patients resided in Tehran (66.67%), Isfahan (22.22%), and Ahwaz (11.11%), and none of them suffered from any other predisposing diseases (Table 1).

The environmental strains (n=102) were shown in Table 2. Nucleotide sequences of all isolates were deposited in GenBank under the accession numbers: KY788018–KY788126 and MF422634–MF422636.

**Table 2:** Environmental dematiaceous fungi isolated from different regions of Iran

City (no.)	Source (no.)	Taxon name (no.)	ITS rDNA Accession nr.
Ahwaz (10)	Air (5)	*Alternaria tenuissima* (2), *Cladosporium cladosporioides* (2), *Didymella glomerata* (1)	KY788031 & KY788032, KY788051 & KY788052, KY788126
	Soil (3)	*Alternaria alternata* (1), *C. cladosporioides* (1), *Aureobasidium pullulans* (1)	KY788027, KY788053, KY788107
	Plant (1)	*Alternaria terricola* (1)	KY788085
	Organic debris (1)	*A. terricola* (1)	KY788084
Bushehr (29)	Air (11)	*A. alternata* (2), * Cladosporium sphaerospermum* (2), *Curvularia hawaiiensis* (2), * A. tenuissima* (1), *Alternaria malorum* (1), *A. terricola *(1), *Drechslera dematioidea* (1), *Embellisia astragali* (1)	KY788020 & KY788021, KY788061 & KY788062, KY788103 & KY788104, KY788029, MF422634, KY788072, KY788111, KY788122,
	Soil (10)	*Alternaria chlamydospora* (2), *Curvularia spicifera* (2), *A. tenuissima* (1), *Alternaria japonica* (1),* A. terricola* (1), *C. cladosporioides* (1), *C. sphaerospermum* (1), *D. dematioidea* (1)	KY788046 & KY788047, KY788098 & KY788099, KY788035, KY788039, KY788073, KY788054, KY788125, KY788112
	Plant (4)	*A. terricola* (2), *C. cladosporioides* (1), *Neoscytalidium dimidiatum* (1)	KY788070 & KY788071, KY788058, KY788094
	Wood (4)	*C. cladosporioides* (1), *A. tenuissima* (1), *C. cladosporioides* (1), *N. dimidiatum* (1)	KY788059, KY788028, KY788055, KY788093
Isfahan (18)	Air (8)	*A. alternata* (2), *Alternaria sp.* (2), *C. cladosporioides* (2), *C. hawaiiensis* (2)	KY788025 & KY788124, KY788044 & KY788045, KY788048 & KY788050, KY788105 & KY788106,
	Soil (5)	*A. tenuissima* (2), *Alternaria sp.*(1), *C. cladosporioides* (1), *Ochroconis constricta* (1)	KY788036 & KY788037, KY788043, KY788049, MF422635
	Plant (3)	*A. terricola* (2), *N. dimidiatum* (1)	KY788087 & KY788088, KY788096
	Wood (2)	*A. terricola* (1), *N. dimidiatum* (1)	KY788086, KY788095
Shiraz (37)	Air (14)	*D. glomerata* (4), *D. dematioidea* (2), *A. alternata* (2), *A. tenuissima* (1), *A. malorum* (1), *A. terricola* (1), *A. pullulans* (1), *Ochroconis species* (1), *E. phaeomuriformis* (1),	KY788115, KY788116, KY788117 & KY788118, KY788113 & KY788114, KY788019 & KY788022, KY788034, KY788041, KY788077, KY788109, MF422636, KY788120
	Soil (11)	*A. alternata* (2), *C. sphaerospermum* (2), *N. dimidiatum* (2), *A. tenuissima* (1), *Alternaria rosae* (1), *A. terricola* (1), *C. spicifera* (1), *A. pullulans* (1)	KY788018 & KY788024, KY788065 & KY788066, KY788089 & KY788090, KY788033, KY788038, KY788079, KY788100, KY788110
	Plant (3)	*Alternaria sp.* (1), *A. terricola* (1), *C. sphaerospermum* (1)	KY788042, KY788082, KY788064
	Wood (5)	*A. terricola* (3), *C. sphaerospermum* (2)	KY788078, KY788080 & KY788081, KY788063 & KY788069
	Organic debris (4)	*C. sphaerospermum* (2), *Ascochyta rabiei* (1), *A. terricola* (1)	KY788067 & KY788068, KY788119, KY788083
Yasuj (8)	Air (1)	*C. spicifera* (1)	KY788101
	Soil (4)	*A. terricola* (2), *A. alternata* (1), *A. tenuissima* (1)	KY788074 & KY788075, KY788026, KY788030
	Plant (1)	*C. cladosporioides* (1)	KY788056
	Wood (1)	*A. terricola* (1)	KY788076
	Organic debris (1)	*C. cladosporioides* (1)	KY788057

**Figure 1:**
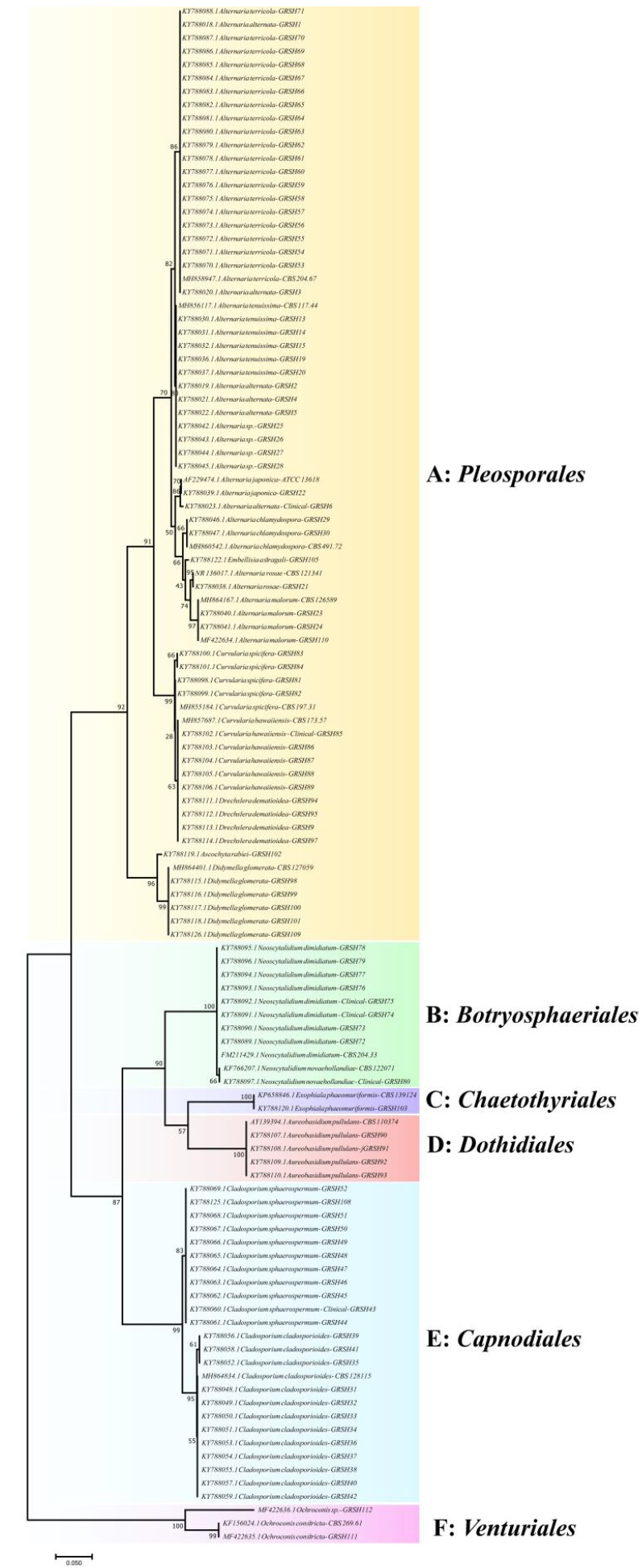
Phylogenetic analysis of black fungi species based on the analysis of ITS sequences. The evolutionary history was inferred using the Maximum likelihood method based on the Tamura–Nei model. A: *Pleosporales*, B: *Botryosphaeriales*, C: *Chaetothyriales*, D: *Dothidiales*, E: *Capnodiales*, F: *Venturiales*

Phylogenetic analyses of ITS sequences of the isolated black fungi revealed six orders, namely *Pleosporales*, *Capnodiales*, *Dothidiales*, *Chaetothyriales*, *Botryosphaeriales*, and *Venturiales* (Figure 1). Closely related genera, such as *Alternaria *, *Curvularia*, *Drechslera *, and *Didymella* species formed well-supported clades with a bootstrap value of 92% while *Neoscytalidium * and Exophiala clustered with a bootstrap value of 100% (Figure. 1).

Remarkably, species of the order *Pleosporales* clustered into two clades: Clades 1 and 2. Clade 1 consisted of strains of *Alternaria *, *Curvularia*, and *Drechslera * species while Clade 2 included *Didymella* as a separate species. The phylogenetic tree revealed *Curvularia* and *Drechslera * in Clade 1, forming a sub-clade closely related to *Alternaria * species. Phylogenetic analysis showed that *Cladosporium* species belonged to the branch *Capnodiales*, forming a single group closely related to *Dothidiales* order (Figure. 1).

In the tree constructed based on the ITS rDNA region, strains of Aureobasidium section *Dothidiales* were located next to the *Chaetothyrials * and *Capnodiales* sections. Member species of *Ochroconis* belonging to the *Venturiales* order branched far away from all the other orders of black fungi.

Table 3 shows the comparison between dematiaceous strains based on the number of differences in the nucleotide sequences. A sequence difference count matrix between these strains ranged from 1 to 464 nucleotides with the largest distance being observed between an *Ochroconis* species and *C. sphaerospermum*. Meanwhile, intra-species differences were found within different strains of *A. alternata*, *A. alternata*, *Alternaria tenuissima* *Curvularia* *spicifera, A. pullulans, C. hawaiiensis, N., dimidiatum,* *Alternaria terricola*, *Alternaria chlamydospora*, *Didymella glomerata*, and *Drechslera dematioidea* by 0-59, 0-22, 0-4, 0-4, 0-3, 0-2, 0-2, 0-2, 0-2, 0-1, and 0-1 nt, respectively (Table 4). Lack of intra-species sequence was observed in *A. malorum* and *C. sphaerospermum* (Table 4). 

**Table 3:** Sequence variation based on pairwise sequence comparison of rDNA genes of dematiaceous fungi.

Taxa (Accession nr)																							
*Alternaria. alternata* (KY788018)	ID																						
*Alternaria. alternata* (KY788023)	47	ID																					
*Alternaria. alternata* (KY788031)	34	63	ID																				
*Alternaria rosae* (KY788038)	73	43	78	ID																			
*Alternaria japonica* (KY788039)	49	12	57	42	ID																		
*Alternaria malorum* (KY788041)	85	50	83	28	46	ID																	
*Alternaria species* (KY788042)	33	62	1	77	56	82	ID																
*Alternaria chlamydospora* (KY788046)	66	28	68	34	33	41	69	ID															
*Cladosporium cladosporioides* (KY788059)	203	238	209	246	242	250	209	240	ID														
*Cladosporium sphaerospermum* (KY788069)	233	266	238	274	270	276	238	268	61	ID													
*Alternaria terricola* (KY788070)	4	46	32	70	47	81	31	63	206	236	ID												
*Neoscytalidium dimidiatum* (KY788094)	197	211	195	211	215	207	195	216	212	234	195	ID											
*Neoscytalidium novaehollandiae* (KY788097)	195	211	194	213	214	208	194	217	213	235	193	4	ID										
*Curvularia spicifera* (KY788100)	114	141	109	138	143	144	110	139	208	236	113	214	213	ID									
*Curvularia hawaiiensis* (KY788104)	114	141	111	138	143	144	112	139	209	237	113	215	214	13	ID								
*Aureobasidium pullulans* (KY788110)	185	202	188	21	202	212	187	205	214	240	187	167	165	215	21	ID							
*Drechslera dematioidea* (KY788111)	114	141	110	138	14	143	111	139	207	235	113	213	212	17	4	217	ID						
*Didymella glomerata* (KY788115)	121	151	117	159	145	165	117	154	212	235	120	202	204	126	126	209	126	ID					
*Ascochyta rabiei* (KY788119)	118	147	115	156	14	161	114	152	202	224	117	198	200	125	126	202	126	26	ID				
*Exophiala phaeomuriformis* (KY788120)	269	277	268	27	278	277	267	279	281	303	272	257	256	275	279	243	279	274	269	ID			
*Embellisia astragali* (KY788122)	70	35	74	36	32	48	75	32	245	273	67	217	220	145	145	215	146	156	153	282	ID		
*Ochroconis constricta* (MF422635)	366	358	364	353	35	344	365	355	389	413	368	345	346	374	370	374	370	374	371	361	352	ID	
*Ochroconis species* (MF422636)	421	409	413	411	408	403	414	406	436	464	421	410	408	422	423	425	422	425	425	429	403	242	ID

**Table 4:** Intra-species variation based on pairwise sequence comparison of the rDNA genes of dematiaceous fungi.

Species	Numbers	Range of intra-species difference (base pair)
*Alternaria alternata*	7	0-59
*Alternaria tenuissima*	6	0-4
*Alternaria malorum*	3	0
*Alternaria species*	4	0
*Alternaria chlamydospora*	2	0-2
*Neoscytalidium dimidiatum*	6	0-2
*Aureobasidium pullulans*	4	0-3
*Curvularia hawaiiensis*	5	0-2
*Cladosporium sphaerospermum*	11	0
*Cladosporium cladosporioides*	12	0-22
*Alternaria terricola*	19	0-2
*Didymella glomerata*	5	0-1
*Drechslera dematioidea*	4	0-1
*Curvularia spicifera*	4	0-4

## Discussion

With increasing recognition of the crucial role of fungi in animal and human infections, diagnostic laboratories are expected to be able to quickly detect and accurately identify fungal pathogens to ensure timely and appropriate therapy for infected patients [ 30 ]. Lack of pigment or poor sporulation, inter-specific similarities, intra-specific diversity, and variation in growth requirements are some of the features that may influence the precise identification of species. Hence, molecular methods are necessary to distinguish and/or re-classify similar and complex taxa of dematiaceous fungi and discover novel and undescribed species [ 9 - 11 ]. Many authors have demonstrated the usefulness of ITS rDNA for species delineation in dematiaceous fungi as the region usually enables discrimination between closely related species [ 3 , 21 , 22 ]. Therefore, in the present study, ITS sequences were utilized for identification as well as phylogenetic analysis of the isolated dematiaceous fungi.

Based on the findings, *Alternaria * was the predominant genus in both environmental and clinical samples. The results of the present study are compatible with those of previous reports [ 31 ], [ 32 ]. However, in a study performed by Parham et al., *Ulocladium* species were the predominant fungi among all of the dematiaceous fungi, and this finding is not in line with that of the present research [ 33 ]. The differences may be due to the source of samples, methods, and other reasons [ 3 ]. 

In the present study, *Alternaria species, C. cladosporioides,* and *C. sphaerospermum* were the most commonly observed strains. Differentiation of some species of black fungi, such as *Cladosporium* species and *A. malorum*, which are common in both clinical and environmental settings, remains difficult. In this study, isolates identified as *Cladosporium* species according to the morphological characteristics were recognized as *A. malorum* based on DNA sequencing. Sequence difference count matrix based on nucleotide pairwise comparison of the ITS region provided evidence showing that this locus was more useful than morphological features for discrimination of these two species.

In a study conducted by Abliz et al., who used the D1/D2 domain for the identification of black fungi, some species of the genus *Cladosporium* were found to have identical or highly similar sequences with substitutions only at one or two positions [ 8 ]. For such species, ITS-rDNA with greater nucleotide variation has a higher potential for discriminating between species than the D1/D2 domain [ 34 , 35 ]. In recent years, DNA-based studies have shown multiple non-monophyletic genera within the *Alternaria * complex that do not always associate with species groups based on morphological characteristics. 

In the present study, phylogenetic relationships constructed based on sequences of the ITS region from *Alternaria* isolates and other *Pleosporaceae* (*Ulocladium* species, *Embellisia* species) show the formation of a distinctive clade consisting of *A. alternata*, *A. tenuissima* (*Alternaria* section), *A. malorum* (*Chalastospora* section), *Alternaria* *japonica* (*Japonica* section), *A. chlamydospora* (*Phragmosporae* section), *A. terricola* (*Ulocladioides* section) and *Embellisia astragali* (*Embellisioides* section) supported by a bootstrap value of 91%. Results of the present study supported previous observations of the polyphyletic and paraphyletic relationship between *Alternaria * and the related taxa of *Ulocladium* and *Embellisia*. 

Among our samples, *C. hawaiiensis, C. spicifera,* and *D. dematioidea* were also isolated. Although the genus *Curvularia* can easily be distinguished from *Bipolaris* and *Drechslera * species by sequence analysis, there has been some difficulty in distinguishing them due to their conidial shape, size, and septation. The *C. hawaiiensis, C. spicifera*, and *D. dematioidea* went together in our analysis, supported by a bootstrap value of 99%. Moreover, the closely related species, *D. glomerata* and *Ascochyta rabiei* formed a separate clade with bootstrap values of 100 (Figure 1). 

In this study, *Exophiala phaeomuriformis* (belonging to the order *Chaetothyriales*) and two *Ochroconis* species were isolated. *Ochroconis* species cause diseases in vertebrate animals and occasionally humans [ 36 ]. Phylogenetic analysis based on sequences of the ITS region from *Ochroconis* isolates indicated that they stand on a separate branch (Figure 1). The *N. dimidiatum* and*N. novaehollandiae* were also among the isolates in the present study. The *N. dimidiatum* is phylogenetically closely related to *N. novaehollandiae* [ 37 ]. Results of the present research are consistent with those of a study performed by Polizzi et al. [ 38 ] which indicated that both species fell into the same clade supported by a bootstrap value of 100%. 

Sequence variation between strains of dematiaceous fungi led to the observation of clusters with different sections of species. While intra-species sequence diversity of dematiaceous fungi, including *A. malorum*, *Alternaria * species, *C. sphaerospermum*, *D. glomerata*, and *D. dematioidea* was low, inter-species nucleotide diversity between most species was quite high. These data advocated that the ITS domain is appropriately variable to be applicable to the identification of several taxa of dematiaceous fungi. The phylogenetic trees constructed from the sequence data revealed that species in the same order segregated into the same cluster.

It was recognized that ITS rDNA sequences do not always provide ample information to differentiate species in the genus *Alternaria *. The collected data showed a small degree of polymorphism between clinical and environmental isolates as well as a quite low degree of polymorphism within isolates of the same group (non-clinical or clinical group). This might indicate that environmental strains can be a source of human infection. Therefore, more studies on clinical isolates are critical to investigate this issue in greater detail. 

## Conclusion

In conclusion, identification of black fungi on the basis of morphological characteristics alone is unreliable for the correct determination of species. The ITS sequences were evaluated to be applicable for the identification of several taxa of black fungi. However, for *Alternaria * species, larger rDNA regions or other gene targets are critical for a better understanding of the taxonomy of this diverse group of fungi.

## Acknowledgments

This study was supported by Tehran University of Medical Sciences, Tehran, Iran (grant No. 94-172 01-27-28538) and Teikyo University of Medical Mycology, Tokyo, Japan.

## Authors’ contribution

Gh.Sh. and H.M. conceptualized and supervised the study. S.N-S., N.J., S.K., M.N., K.M., and M.Gh. provided resources. Gh.Sh. performed the research. Gh.Sh. and B.A. performed formal analysis. Gh.Sh. and H.M. prepared the original draft. H.B., K.M., and K.S. review the draft and edited it. All authors commented on the manuscript. All authors read and approved the final manuscript.

## Conflicts of interest 

The authors declare that they have no conflicts of interest.

## Financial disclosure

No financial interests related to the material of this manuscript have been declared.
